# Enhanced accuracy with Segmentation of Colorectal Polyp using NanoNetB, and Conditional Random Field Test-Time Augmentation

**DOI:** 10.3389/frobt.2024.1387491

**Published:** 2024-08-09

**Authors:** Muhammad Sajjad Hussain, Umer Asgher, Sajid Nisar, Vladimir Socha, Arslan Shaukat, Jinhui Wang, Tian Feng, Rehan Zafar Paracha, Muhammad Ali Khan

**Affiliations:** ^1^ Department of Computer Science, Sir Syed (CASE) Institute of Technology, Islamabad, Pakistan; ^2^ Laboratory of Human Factors and Automation in Aviation, Department of Air Transport, Faculty of Transportation Sciences, Czech Technical University in Prague (CTU), Prague, Czechia; ^3^ School of Interdisciplinary Engineering and Sciences (SINES), National University of Sciences and Technology (NUST), Islamabad, Pakistan; ^4^ Department of Mechanical and Electrical Systems Engineering, Faculty of Engineering, Kyoto University of Advanced Science, Kyoto, Japan; ^5^ Department of Information and Communication Technology in Medicine, Faculty of Biomedical Engineering, Czech Technical University in Prague, Prague, Czechia; ^6^ Department of Computer and Software Engineering, College of Electrical and Mechanical Engineering (CoEME), National University of Sciences and Technology (NUST), Islamabad, Pakistan; ^7^ Institute for Brain Research and Rehabilitation, South China Normal University, Guangzhou, China; ^8^ Department of Physical Education, Physical Education College of Zhengzhou University, Zhengzhou, China; ^9^ Department of Mechanical Engineering, College of Electrical and Mechanical Engineering (CoEME), National University of Sciences and Technology (NUST), Islamabad, Pakistan; ^10^ School of Mechanical and Manufacturing Engineering (SMME), National University of Sciences and Technology (NUST), Islamabad, Pakistan

**Keywords:** colonoscopy, conditional random field, test-time augmentation, lightweight deep learning models, polyp segmentation, colorectal cancer

## Abstract

Colonoscopy is a reliable diagnostic method to detect colorectal polyps early on and prevent colorectal cancer. The current examination techniques face a significant challenge of high missed rates, resulting in numerous undetected polyps and irregularities. Automated and real-time segmentation methods can help endoscopists to segment the shape and location of polyps from colonoscopy images in order to facilitate clinician’s timely diagnosis and interventions. Different parameters like shapes, small sizes of polyps, and their close resemblance to surrounding tissues make this task challenging. Furthermore, high-definition image quality and reliance on the operator make real-time and accurate endoscopic image segmentation more challenging. Deep learning models utilized for segmenting polyps, designed to capture diverse patterns, are becoming progressively complex. This complexity poses challenges for real-time medical operations. In clinical settings, utilizing automated methods requires the development of accurate, lightweight models with minimal latency, ensuring seamless integration with endoscopic hardware devices. To address these challenges, in this study a novel lightweight and more generalized Enhanced Nanonet model, an improved version of Nanonet using NanonetB for real-time and precise colonoscopy image segmentation, is proposed. The proposed model enhances the performance of Nanonet using Nanonet B on the overall prediction scheme by applying data augmentation, Conditional Random Field (CRF), and Test-Time Augmentation (TTA). Six publicly available datasets are utilized to perform thorough evaluations, assess generalizability, and validate the improvements: Kvasir-SEG, Endotect Challenge 2020, Kvasir-instrument, CVC-ClinicDB, CVC-ColonDB, and CVC-300. Through extensive experimentation, using the Kvasir-SEG dataset, our model achieves a mIoU score of 0.8188 and a Dice coefficient of 0.8060 with only 132,049 parameters and employing minimal computational resources. A thorough cross-dataset evaluation was performed to assess the generalization capability of the proposed Enhanced Nanonet model across various publicly available polyp datasets for potential real-world applications. The result of this study shows that using CRF (Conditional Random Fields) and TTA (Test-Time Augmentation) enhances performance within the same dataset and also across diverse datasets with a model size of just 132,049 parameters. Also, the proposed method indicates improved results in detecting smaller and sessile polyps (flats) that are significant contributors to the high miss rates.

## 1 Introduction

Colorectal cancer (CRC) is the third most prevalent cancer and is the second most common cause of death worldwide, contributing to approximately 8% of all cancer-related deaths globally (Jemal et al., 2010; Bray et al., 2018). Timely detection and resection of premalignant polyps play a crucial role in lowering the risk and mortality of colorectal cancer. Colorectal Polyp is an abnormal growth on the inner lining of the colon and rectum. Approximately 95% of colorectal cancer cases originate from adenomatous polyps (Aarons et al., 2014). A study reports a miss rate of 17.24% of colorectal polyps, with 98.4% of missed polyps being <10 mm in diameter, 98% being sessile or flat in appearance, and 29.8% at the ascending colon (Lee et al., 2017). Multiple invasive and non-invasive tests exist for screening for CRC. Still, colonoscopy, an invasive technique involving invasive examination of colonic mucosa and biopsies of the lesion, is the gold standard (Uraoka et al., 2015) with a specificity of 73.2% and sensitivity of 92.5% (Issa and NouredDine, 2017). Recent research indicates a 67% decrease in the risk of death from colorectal cancer (CRC) (Doubeni et al., 2018) associated with colonoscopy. Many polyps are missed during colonoscopy due to the older age of patients, smaller adenoma size, existence of concurrent protruding adenoma, inadequate colon cleansing, insufficient experience of colonoscopists, structure of the colon, and withdrawal time of <6 min (Xiang et al., 2014). Only a tiny fraction of video frames contains polyps during endoscopy, while the rest are not informative. Hence, there exists a requirement for an automated computer-assisted diagnosis system that detects and segments these overlooked polyps in real time during colonoscopy screening with high accuracy and precision.

A computer-aided diagnosis (CADx) system designed to segment polyps can enhance monitoring and diagnostic proficiency by elevating performance and minimizing manual intervention. Furthermore, it can potentially mitigate segmentation errors compared to subjective approaches. Integrating such systems not only alleviates the workload of medical professionals but also enhances the efficiency of clinical workflows. Developing a well-generalized model represents a substantial advancement toward clinical systems that meet acceptable standards. Cross-dataset evaluation is vital for assessing the model’s efficacy on unseen polyps from various sources, affirming its capability to generalize effectively. Computer-aided systems are generally categorized into two distinct groups: handcrafted and deep learning techniques. Earlier studies focused on using handcrafted descriptors-based features to obtain intrinsic features of polyps like shape, colour, edges, and texture determined by researchers and passed to a classifier to distinguish lesions from surrounding tissues (Karkanis et al., 2003; Ameling et al., 2009). However, conventional Machine Learning approaches, that rely on handcrafted descriptor features suffers from low performance (Bernal et al., 2012). Deep learning has provided new opportunities to address challenges like excessive or insufficient lighting, bleeding, smoke, and reflections (Bodenstedt et al., 2018). For the automated segmentation of medical images (Litjens et al., 2017), Convolutional Neural Networks (CNNs) have exhibited cutting-edge performance.

Some studies suggested that enhancing the performance of existing models is possible by strategically applying post-processing techniques (Jha et al., 2019; Ibtehaz and Rahman, 2020). A famous image segmentation architecture, U-Net was proposed by (Ronneberger et al., 2015), comprising analysis and synthesis path. Various variants of U-net architectures were developed (Milletari et al., 2016; Alom et al., 2018; Zhou et al., 2018; Huang et al., 2020; Qin et al., 2020) for biomedical image segmentation to achieve better results. Later, DoubleU-Net (Jha et al., 2020) was presented for segmenting polyps in colonoscopy images. The DoubleU-Net architecture comprises two UNETS that achieve exceptional performance, outperforming current benchmarks. Similarly, ResUNet++ (Jha et al., 2019) enhances the conventional U-Net framework performance by incorporating several blocks, including the squeeze-and-excite block (S&E block) (Hu et al., 2018), atrous spatial pyramid pooling (ASPP), attention block (Li et al., 2018), and residual block (He et al., 2016). Vanishing and exploding gradient are among the problems that the residual block helps to mitigate, especially when the neural network’s depth grows. Meanwhile, feature map calibration is done by the S&E block by using convolution to account for channel importance.

As the neural network’s depth grows, obtaining detailed information becomes challenging due to reduced feature map size. To overcome this challenge, ResUNet++ employs ASPP, which aids in preserving detailed information and facilitating precise predictions at the pixel level (Jha et al., 2021). enhanced ResUNet++ performance with conditional random field (CRF) Alam et al., 2019) and test-time augmentation (TTA) SOTA (Moshkov et al., 2020). A probabilistic approach called CRF makes it easier to predict pixel labels with accuracy, whereas TTA takes the average of the anticipated values of enhanced images. For the Kvasir-SEG dataset, the suggested model outperformed the current ResUNet++ by 4%, with a Dice coefficient of 85% or above (Srivastava et al., 2021). introduced another model named MSRF-Net, designed explicitly for segmenting polyps of various sizes. MSRF-Net comprises an encoder, a shape stream (Sun et al., 2020), an MSRF-sub network, and a decoder. Two S&E blocks are combined by the encoder, which also connects the output to the MSRF-sub network. Dual-scale dense fusion blocks in several sizes make up the MSRF-sub network. These blocks manage the encoder’s feature maps, transfer data between scales, maintain low-level features, and enhance information flow while maintaining resolution. Next, the shape stream block is traversed by the feature map, which improves spatial accuracy. The MSRF-sub network is connected to a triple attention block in the decoder, and a residual connection is used in the previous decoder output. Inside the decoder, the S&E block figures out each channel scale. With superior segmentation performance, MSRF-Net excels in shaping and classifying polyps of different sizes. Nevertheless, it performs not as well in situations where the images have low contrast.

So, deep learning has proven to be highly effective in segmenting medical images, but it demands a significant amount of representative data. In healthcare, datasets are complex to collect due to privacy concerns, standardization challenges, high image acquisition costs, lack of annotated and high-quality images for training (Jha et al., 2019), and the considerable variation of images among patients (Wang et al., 2018). Hence, obtaining a medical dataset is a challenging task. Thus, to solve a semantic segmentation task, a compelling and viable approach is to reuse ImageNet pre-trained encoders (Chen et al., 2018). Also, deep learning-based architectures tend to be complex and computationally expensive, and their training requires high-end GPUs (Jha et al., 2019; Jha et al., 2020; Jha et al., 2021). Furthermore, the real-time lesion segmentation task needs to be addressed. Although there has been some recent advancement in real-time colonoscopy image segmentation, private datasets are primarily employed for experimentation (Yamada et al., 2019; Lee et al., 2020; Bardhi et al., 2021). It is difficult to evaluate new methods on proprietary datasets and raise the benchmark. Thus, benchmarks on publicly available datasets are needed to bridge the research gap and develop a model suitable for clinical use. One should be very careful in the developmental phase to integrate deep learning models into real-time applications, such as the segmentation of polyps into specific hardware devices (e.g., medical capsule robots).

An efficient model should have low hardware requirements, be easy to train, and involve less trainable parameters. Our study shows more work needs to be done in developing lightweight models. Developing efficient semantic segmentation methods for real-time applications requires a lightweight Convolutional Neural Network (CNN) model. Usually, these models require less memory and are computationally efficient, primarily deployed in mobile applications (Kim et al., 2015). A lightweight model is essential for efficient real-time predictions in resource-limited clinical settings. In the literature, few studies focused on developing lightweight CNN-based models for the segmentation of colonoscopy images (Wang et al., 2019). proposed a lightweight LEDNet architecture that uses a pre-trained encoder using Resnet50. An attention pyramid network (APN) was applied in the decoder stage to reduce model complexity further. SqueezeNet (Iandola et al., 2016) performs excellently in multiplication accumulation and memory use with reduced model size. A very efficient and lightweight encoder and decoder architecture, Nanonet was proposed by (Jha et al., 2021), using MobileNetV2 (Sandler et al., 2018) as a pre-trained encoder that can be incorporated with any device because of fewer trainable parameters. In Nanonet, three models were presented, Nanonet A, B, and C, with trainable parameters (235,425, 132,049, and 36,561). For our work, we have used NanonetB for experimentation.

More realtime, and generalized polyp segmentation models are clearly needed, based on the results of previous research. (Jha et al., 2021). utilized post-processing techniques to enhance performance of ResUnet++ which does not use any pre-trained weights. In our work, we used Nanonet as backbone architecture which uses a pre-trained encoder MobileNetV2 and custom decoder is built accordingly. Furthermore, impact of post-processing techniques on lightweight models using a pre-trained encoder has never been utilized in literature. Thus, we aim to develop a more robust, generalized, and lightweight model that requires less memory and computational resources and can easily be integrated with colonoscopic hardware devices. By applying a variety of techniques, such as data augmentation, conditional random field (CRF), and test-time augmentation (TTA), the proposed model significantly improves the accuracy of Nanonet-B. The results obtained are promising and outperform other state-of-the-art methods like Nanonet (Jha et al., 2021), ResUnet (Hou et al., 2016), ResUnet++ (Jha et al., 2019), HarDNet-MSEG’2021 (Huang et al., 2021), UNeXt ’2022) (Valanarasu and Patel, 2022), and TransNetR’ (Jha et al., 2023). To increase the training data, we have performed considerable data augmentation. We conducted a thorough evaluation by incorporating additional metrics and provided rationale for including conditional random field (CRF) and test-time augmentation (TTA) in the proposed model. Additionally, we stressed the significance of resolving issues associated with the misidentification of sessile and flat polyps. The proposed combined methodology demonstrated high efficiency in detecting overlooked polyps, showcasing its potential importance in clinical settings. Additionally, to achieve the goal of generalizability, we tested and trained the model using images from other sources, underscoring the importance of cross-dataset evaluation. In summary, the main contribution of this paper is as follows:

a. Novel lightweight and real time Enhanced Nanonet models (CRF, TTA and their combination) with few parameters using NanonetB to segment colonoscopy images are proposed for better performance and generalizability. Extensive data augmentation, post-processing techniques like conditional random field (CRF), and test-time augmentation (TTA) are applied to enhance colorectal polyp segmentation results.
b. The proposed approach shows promising results when compared to other advanced complex deep learning algorithms like U-Net, DoubleUnet, ResUnet, and ResUnet++, Nanonet (A, B, and C), HarDNet-MSEG, UNeXt, and Transnet on six different datasets Kvasir-SEG (Jha et al., 2019), Endotect Challenge (Hicks et al., 2021), Kvasir-Instrument (Jha et al., 2020), CVC-ClinicDB (Bernal et al., 2015), CVC-ColonDB (Bernal et al., 2012), and CVC-300 (Sánchez et al., 2017). The proposed model with few parameters outperforms complex deep learning models regarding computation, speed, parameter use (size), and performance metrics.
c. For sessile and smaller polyps that are mostly missed during colonoscopy (Zimmermann-Fraedrich et al., 2019), the proposed model achieves a promising segmentation result, a vital strength of our work.
d. In medical clinical practice, models that demonstrate generalizability are crucial for addressing diverse patient populations. Our focus is on exploring generalizability, a dimension that has received limited attention in the community thus far. So, we trained the model on Kvasir-SEG, testing and comparing the results across three distinct polyp datasets that were previously unseen.
e. The proposed enhanced NanonetB model can be integrated into any real-time environment, such as colonoscopy and mobile devices, due to the improved accuracy of the proposed model with considerably fewer parameters.

This paper is structured as follows: Section 2 outlines the proposed methodology. Section 3 describes the material and methods being utilized. In Section 4, we present experimental findings along with a comparison with other models. In Section 5, qualitative and quantitative results are discussed explicitly, along with the conclusion.

## 2 Proposed methodology

**FIGURE 1:**
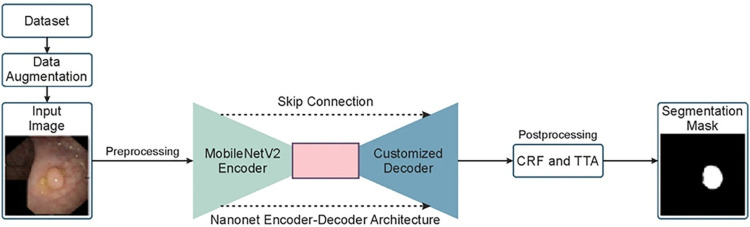
Overview of the proposed Enhanced Nanonet model.

Figure 1 depicts a comprehensive summary of the research. Our proposed Enhanced Nanonet models uses Nanonet (Jha, Tomar, et al., 2021) architecture as a backbone, an encoder-decoder approach. Datasets are subjected to substantial data augmentation to improve robustness and produce more adaptive systems. By utilizing Conditional Random Field (CRF) and Test-Time Augmentation (TTA), the proposed approach improves NanonetB’s overall prediction performance. All the improvements are validated and performed comprehensive evaluations using six distinct datasets: Kvasir-SEG, Endotect Challenge 2020, Kvasir-instrument, CVC-ClinicDB, CVC-ColonDB, and CVC-300.

### 2.1 Network architecture

**FIGURE 2:**
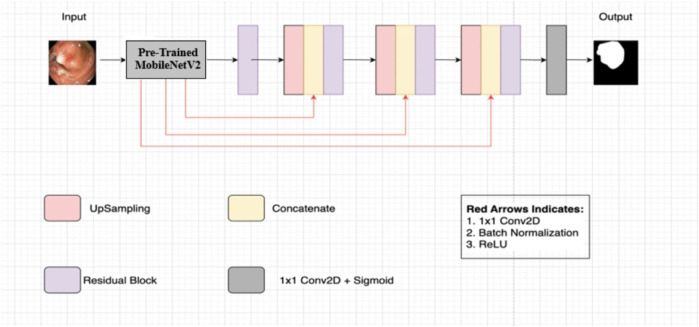
Nanonet architecture (Jha et al., 2021).

Figure 2 illustrates the Nanonet architecture, based on an encoder-decoder approach (Jha et al., 2021). This architecture leverages a pre-trained encoder followed by three decoder blocks, with a modified residual block acting as a bridge between the encoder and decoder.

#### 2.1.1 Encoder

The encoder employs pre-trained ImageNet models (Deng et al., 2010) using transfer learning, which accelerates model convergence and enhances performance compared to models without pre-training. Specifically, Nanonet uses MobileNetV2 with ImageNet weights (Deng et al., 2010) in the encoder block. MobileNetV2 is chosen for its fast convergence and reduced computational cost. The encoder receives input images of size 256 × 256 and processes them using inverted residual blocks, which include standard convolution with 3 × 3 kernels and 32 feature channels, ReLU6 activation in the bottleneck layer, and batch normalization. Feature maps are down-sampled progressively using strided convolution, and feature channels are increased gradually.

#### 2.1.2 Modified residual block

The modified residual block serves as a bridge between the encoder and decoder. It takes the output from the encoder and employs bilinear upsampling to restore the spatial dimensions of the feature maps to their original size in the decoding pathway. The feature maps are concatenated with upsampled feature maps using skip connections from the pre-trained encoder. These skip connections help preserve and propagate information between layers, avoiding the vanishing gradient problem and enhancing feature map quality (Drozdzal et al., 2016; Hou et al., 2016).

#### 2.1.3 Decoder

The decoder consists of three blocks that process the concatenated feature maps from the modified residual block. Each decoder block follows the same process, gradually refining the feature maps. Finally, a sigmoid activation function and a 1 × 1 convolutional layer are applied in the network’s final block to complete the segmentation task.

#### 2.1.4 Architectural variants

Nanonet proposes three different architectural variants: NanonetA, NanonetB, and NanonetC, each with distinct feature channel configurations within its decoder blocks. NanonetA uses 32, 64, and 128 feature channels, while NanonetB and NanonetC use progressively fewer feature channels, reducing from 32, 64, and 96 to 16, 24, and 32 (Jha et al., 2021). This reduction in feature channels results in fewer trainable parameters, reducing model complexity and yielding a lightweight model.

#### 2.1.5 Integration of technologies

The novelty of this study lies in the integration of these techniques into a single, cohesive framework specifically designed for real-time polyp segmentation in colonoscopy images. Nanonet integrates elements from various advanced architectures: ResUnet (Hou et al., 2016) provides the backbone structure for our model. Modified Residual Blocks are incorporated to enhance channel interdependencies and allow deeper networks without degradation while maintaining computational efficiency. Additionally, SE-Blocks (Hu et al., 2018) are used to improve feature map quality by re-calibrating channel-wise responses. The U-Net (Ronneberger et al., 2015) contributes to the overall encoder-decoder structure, ensuring robust segmentation performance. By leveraging the strengths of each method and combining them in a lightweight architecture, we achieve superior performance with less computational resources and more generalizability.

### 2.2 Squeeze and excite methodology

Squeeze and excitation, also known as SE-Block (Hu et al., 2018), is one of the channel-wise attention mechanisms that re-calibrate each channel to create a more robust representation for CNNs by highlighting the essential features. By acquiring channel weights based on global spatial information, the SE block simultaneously suppresses feature maps that are not important and increases the sensitivity of better feature maps. The feature maps generated by the convolutional process can only record local information; they cannot access the global information stored by the local receptive field. Thus, comprehensive global information of the feature map from each channel is acquired, and a squeeze method is utilized using Global average pooling, resulting in a feature map with a dimension of B × H × W × C instead of B × 1 × 1 × C. Furthermore, using sigmoid activation, the model can identify non-linear interactions between channels and capture channel-specific dependencies. Excitation is performed to get channel-wise dependencies and learn non-linear dependencies between channels. The SE net exhibits remarkable generalization capabilities across diverse datasets. The SE block and a modified residual block are combined in Nanonet architecture to improve the efficiency of generalization across multiple datasets, thus enhancing the efficiency of the model.

### 2.3 Modified residual block

Training a deeper neural network by simply expanding the CNN layers can hinder the training process due to the vanishing gradient problem during backpropagation (Tan and Lim, 2019). In the first convolution, the original residual block comprises two 3 × 3 standard convolutions alongside batch normalization and ReLu activation. The identity mapping and batch normalization output are added element-wise in the second convolution, followed by another activation function, ReLU. An identity mapping involves applying 1 × 1 standard convolution and batch normalization to the original input. The working principle of ResUnet is illustrated in Eq. 1.

$$yn=F\;\left(xn,Wn\right)+xn$$ (1)

The input is xn , and the residual function is F ( xn , Wn ), followed by a sequence of convolution layers, batch normalization, and ReLu activation. In this work, the residual block undergoes some modifications involving 1 × 1 convolution, followed by 3 × 3 convolution. The number of filters in both convolutions is reduced to ¼, and batch normalization and the ReLU activation function are applied. Afterward, a 3 × 3 convolution operation is used along with batch normalization. Finally, identity mapping is incorporated by performing element-wise addition. In the end, ReLU activation followed by squeeze and excitation block (SE) is applied, improving the features representation by highlighting the important ones.

### 2.4 MobileNetV2

The MobileNetV2 architecture builds upon the MobileNetV1 architecture, incorporating depth-wise separable convolutions as its primary building blocks. MobilenetV2 (Sandler et al., 2018) attains outstanding results across different datasets with fewer parameters. For mobile and embedded devices, MobileNetV2 is specially designed, thus contributing to a more efficient use of computational resources. The proposed Nanonet architecture uses MobileNetV2 ImageNet weights (Deng et al., 2010) as the pre-trained encoder. In contrast to the traditional residual deep neural network, MobileNetV2 uses thin bottleneck layers as input and output of residual blocks. The MobileNetV2 architecture is built on the concept of inverted residual blocks (or structures) with a linear bottleneck. The inverted residual block, inspired by the bottleneck residual block, comprises three consecutive convolutions (1 × 1, 3 × 3, and 1 × 1), each succeeded by a Rectified Linear Unit (ReLU) activation. Unlike the bottleneck block, feature channels are expanded by the first 1 × 1 convolution, while the last 1 × 1 convolution reduces them. The block concludes with an element-wise addition involving identity mapping, distinguishing it as an inverted residual block. MobileNetV2 will learn and filter the image characteristics fed to a network using compact depth-to-depth convolution. Thus, inverted residual blocks will allow the model to converge faster with fewer parameters than a non-pertained network. In the linear bottleneck, linear activation is applied before performing element-wise addition with identity mapping in the last 1 × 1 standard convolution layer.

### 2.5 Conditional random field

**FIGURE 3:**
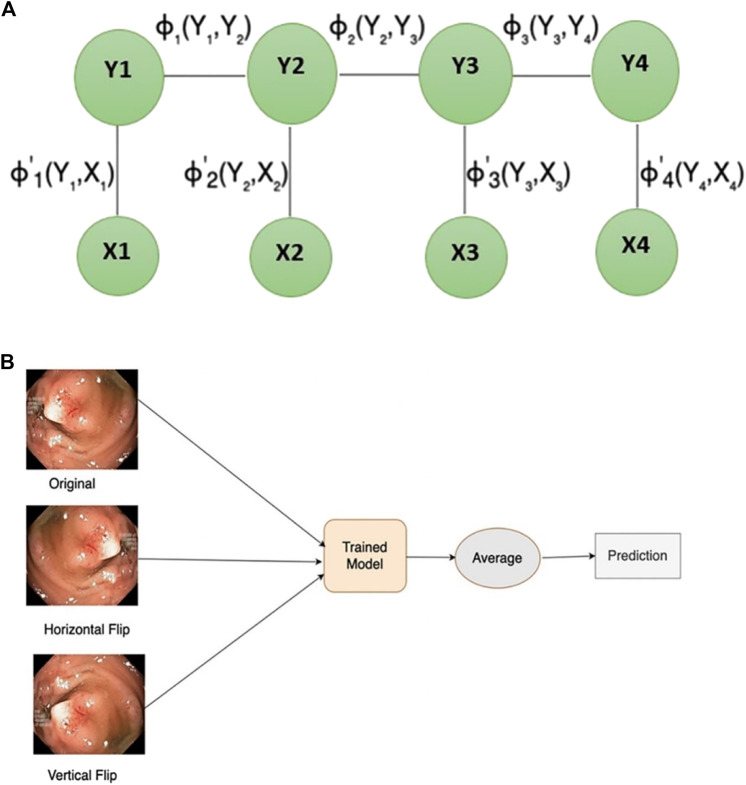
**(A)** Conditional random field structure **(B)** test-time augmentation.

In scenarios when the class labels of various inputs exhibit dependencies (e.g., image segmentation tasks), a conditional random field (CRF) emerges as a notable discriminative modelling approach. CRF (see Figure 3A) with CNN leads to improved performance by modelling the spatial contextual dependencies between the regions. Conditional random fields are employed to obtain effective geometric attributes like region, shape connectivity, and contextual information between the regions (Alam et al., 2019). Thus, incorporating conditional random field (CRF) can enhance the overall segmentation outcomes by contributing more towards capturing the contextual information of the polyps. In this work, dense conditional random forest (CRF) is being utilized to enhance the overall segmentation accuracy on the test dataset.

### 2.6 Test-time augmentation

Data augmentation is an approach utilized to generate new samples from existing ones, mainly applied during model training. During the validation stage, new augmented images are produced from the test dataset using test-time augmentation (TTA) to improve overall prediction performance. TTA (see Figure 3B) has the final output by averaging the model predictions from different augmented images of test input. TTA enhances model performance, requires minimal computational resources using a pre-trained model, eliminates the need for hyperparameter tuning, and allows for parallelized predictions on multiple augmented images. Inspired by the most recent SOTA (Moshkov et al., 2020), this paper uses the vertical and horizontal flip for TTA.

## 3 Materials and methods

Six different datasets for training, testing, and validation of models are used to evaluate enhanced Nanonet architecture. Furthermore, evaluation metrics, hardware implementation details, and data augmentation techniques being employed will be discussed in this section.

### 3.1 Datasets

**FIGURE 4:**
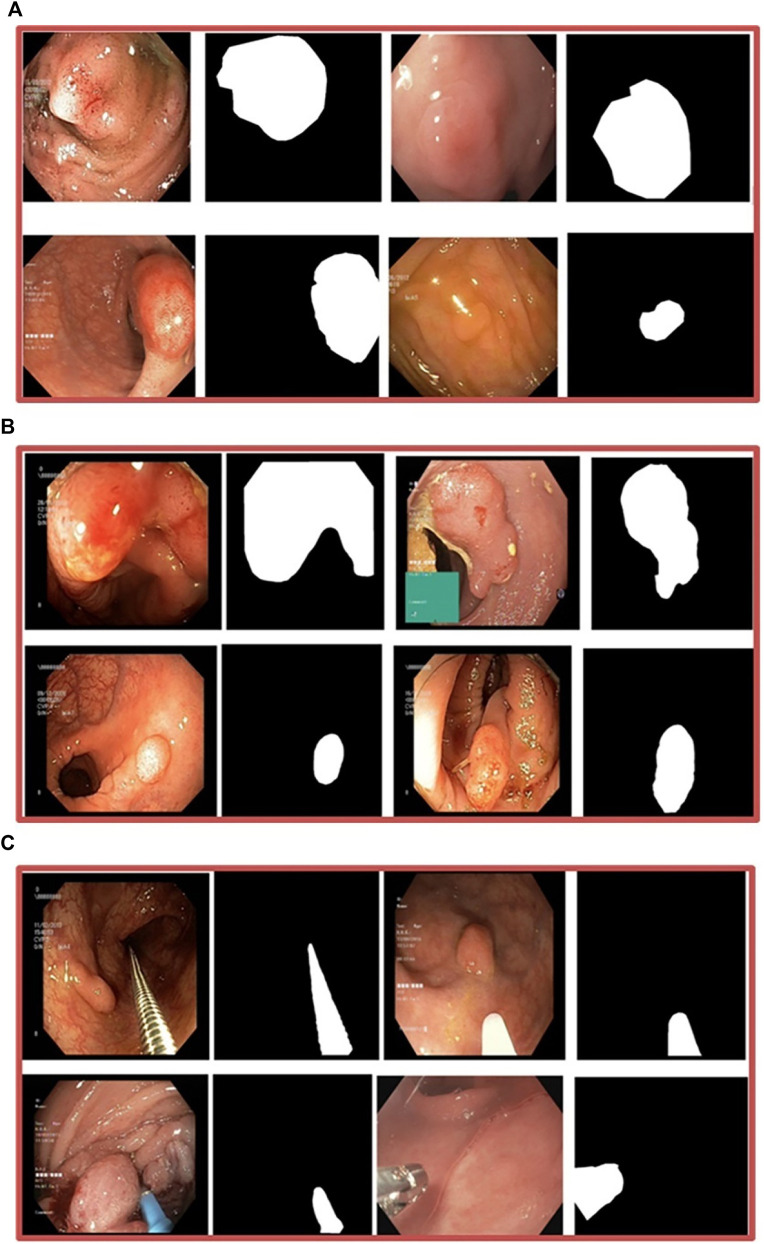
**(A)** Kvasir-SEG dataset, images of polyps and associated ground truth masks **(B)** Endotect Challenge dataset **(C)** Kvasir-Instrument dataset.

Our experiments used six different datasets comprising segmented polyps and corresponding ground truth masks. They exhibit variations, such as differences in the number of images, resolution of images, and the devices employed. Kvasir-SEG (Jha et al., 2019) is one of the three datasets used in Enhanced-Nanonet. It comprises 1,000 polyp images acquired with a high-resolution electromagnetic imaging system and their corresponding annotated ground truth masks segmented by skilled endoscopists. The source of this dataset is from a clinical examination at Bærum Hospital by expert gastroenterologists in Norway, with a resolution varying from 332 × 487 to 1920 × 1,072 pixels. Images of Polyp accompanied by their corresponding ground truth masks are displayed in Figure 4A. “Endotect challenge” is the second dataset which uses Kvasir-SEG as training (Hicks et al., 2021). In the Endotect challenge, they released 200 images to test the participant approaches. Figure 4B displays polyp images from the “Endotect challenge” dataset together with corresponding ground truth masks. The final dataset, Kvasir-Instrument (Jha et al., 2020), consists of 590 photos collected by endoscopists along with the corresponding ground truth labels. Pixel sizes of the images in the dataset range from 720 × 576 to 1,280 × 1,024. The “Kvasir-Instrument” dataset’s polyp images and ground truth masks are shown in Figure 4C. Three distinct datasets, CVC-ClinicDB (Bernal et al., 2015), CVC-ColonDB (Bernal et al., 2012), and CVC-300 (Sánchez et al., 2017), were also utilized for cross-dataset evaluation utilizing the Kvasir-SEG dataset.

### 3.2 Evaluation method

Standard computer vision metrics including Dice Coefficient (DSC), mean Intersection over Union (mIoU), Precision, Recall, Accuracy, Specificity, and Frames-per-second (FPS) are utilized to access the model performance for the polyp segmentation task. The dice coefficient (DSC) and mean intersection over union (mIoU) are the two metrics that are most frequently utilized. The DSC coefficient is used to assess how closely the segmentation results that are generated match the original ground truth. Similarly, the IoU is used to assess the overlap between the output mask and the original ground truth mask of the polyp. In each image, the mIoU calculates the IoU for each class, and the average is acquired across all classes. Although there is a relationship between mIoU and DSC, both metrics are computed to thoroughly examine the outcomes, contributing to a deeper understanding of the results.

Formula of IOU is as follows:

$$IOU=\frac{Area\;of\;Overlap}{Area\;of\;Union}$$ (2)

Eq. 2 illustrates that the area of overlap represents the shared region between two predicted masks, while the Area of Union encompasses the entirety of the areas covered by both masks.

Below is the formula for the Dice Coefficient:

$$DSC=\frac{2\times \left|X\cap Y\;\right|}{\;\left|X\cup Y\;\right|}$$ (3)

Eq. 3 computes the ratio between the shared and the combined area of the two masks, denoted by X and Y.

In polyp segmentation, precision measures the accuracy of identifying pixels as polyp or non-polyp. In contrast, recall measures the percentage of the test image’s total pixels that have been segmented correctly. Precision and recall help assess over-segmentation and under-segmentation levels in polyps image segmentation. A more detailed explanation can be found in (Shamir et al., 2018; Powers and Ailab, 2020). For binary classification systems, another important metric is receiver operating characteristic (ROC) curve analysis to measure performance. Therefore, we calculate metrics like mIoU, DSC, precision, recall, F2, accuracy and ROC to evaluate proposed segmentation models.

### 3.3 Data augmentation

Data augmentation is essential for reducing overfitting and resolving data insufficiency issues, which enhances model performance. The dataset is increased by applying extensive data augmentation techniques on all three different datasets to improve the diversity and generalization of our model. All polyp datasets are divided into 80:10:10 ratio of training, validation, and testing employing random distribution. After splitting, various data augmentation techniques are applied like RandomRotate90, Crop, Vertical Flip, Elastic Transform, Grid Distortion, Optical Distortion, Horizontal Flip, Grayscale, RGBShift, ChannelShuffle, CoarseDropout, GaussNoise. These data augmentation techniques are limited to the use of training data. We resized the validation and testing sets to 256 × 256 for our experiments to reduce computational complexity. While evaluating the Enhanced Nanonet model using TTA, test data was augmented with a horizontal and vertical flip.

### 3.4 Implementation and hardware details

The Enhanced Nanonet model uses Keras (Joseph et al., 2021) and TensorFlow (Abadi et al., 2016) frameworks as the backend. All the tests were performed on Apple M1 MacBook Air with 8 GB of RAM and eight cores. We used the same dataset to perform various experiments with different hyperparameter configurations. This was done to identify the optimal set of hyperparameters for the proposed model. The dataset’s images have been resized to 256 × 256 pixels to optimize RAM usage and minimize training time. This resizing is done to expedite training and better utilization of RAM, and a batch size of 16 is employed because of the model’s limited trainable parameters. The learning rate was first reduced by a factor of 0.1 from its initial value of 1e-4 when the validation loss did not improve for ten consecutive epochs. This adjustment was made to optimize the model’s performance and update parameters slowly. A key element in training a model is the loss function, which measures the difference between predicted and observed values. In segmentation problems, the loss function is categorized into distribution-based, region-based, and boundary-based functions. For binary segmentation problems, a loss function, Dice loss, is used to evaluate how similar two masks are. Computing the F1-score for the original and predicted masks balances precision and recall for the prediction mask. The optimizer is a critical hyperparameter that affects the model’s performance during training by looking for parameters that minimize the loss function. The formula for dice loss is given below:

$$Dice\;Loss=1-\frac{\left(2y\hat{y}+1\right)}{\left(y+\hat{y}+1\right)}$$ (4)

The predicted mask is indicated by ŷ in the Dice loss function (Eq. 4), whereas y represents the actual mask. Only 37 epochs are used to train the Enhanced Nanonet model for the Kvasir-SEG and Endotect datasets with optimizer Nadam and Dice loss as loss function. Additionally, to prevent overfitting, early stopping is used. For the Kvasir-Instrument dataset, the model is trained with nine epochs to reduce the computational time and complexity. Similarly, we evaluated the models’ generalizability by training models on Kvasir-SEG and performed cross-dataset evaluation using three distinct datasets: CVC-ClinicDB, CVC-ColonDB, and CVC-300.

## 4 Experimental results

In this work, Nanonet performance is enhanced using NanonetB by utilizing hyperparameter optimization, CRF, and TTA. Using six publicly accessible datasets: Kvasir-SEG, Endotect Challenge 2020, and Kvasir-instrument, CVC-ClinicDB, CVC-ColonDB, and CVC-300, several experiments were performed out to demonstrate the impact of the proposed Enhanced-Nanonet models. Furthermore, Enhanced-Nanonet (with CRF, TTA and their combination) performance was compared with recent complex deep learning models like ResUnet, ResUnet++, Nanonet (A, B, and C), HarDNet-MSEG, UNeXt, and TransNetR. Results of the proposed model, along with CRF, TTA, and their combined applications, were showcased and compared in this section on the same and cross-dataset scenarios. Although various testing techniques used by different authors make comparisons with methods from the literature difficult, our goal is to evaluate the results of recent studies. The ROC curve assesses the performance of a classification model at a specific threshold. In this case, we have established a probability threshold of 0.5. Tables 1–3 show improved quantitative Enhanced-Nanonet models results along with a comparison with other SOTA computer vision methods. The proposed lightweight Enhanced-Nanonet model generates a good segmentation map on polyps compared to complex deep learning models that are smaller/flat in size or nonpedunculated polyps, which is very important when aiming to develop real-time polyp segmentation system. Table 4 represents cross-dataset generalization results of the proposed Enhanced Nanonet model, with CRF, TTA and their combination on the Kvasir-SEG dataset as training data.

**TABLE 1:** Performance evaluation and comparison on Kvasir-SEG.

Methods	Parameters	DSC	mIoU	Recall	Precision	F2	FPS	Accuracy
ResUNet (GRSL’18)	8,227,393	0.7203	0.6106	0.7602	0.7624	0.7327	-	0.9251
ResUNet++ (ISM’19)	4,070,385	0.7310	0.6363	0.7925	0.7932	0.7478	-	0.9223
Nanonet-A	235,425	0.8227	0.7282	0.8588	0.8367	0.8354	-	0.9456
Nanonet-B	132,049	0.7860	0.6799	0.8392	0.8004	0.8067	-	0.9365
Nanonet-C	36,561	0.7494	0.6360	0.8081	0.7738	0.7719	-	0.9290
HarDNet-MSEG	33.34M	0.8260	0.7459	0.8485	0.8652	0.8358	-	-
UNeXt	1.47M	0.7318	0.6284	0.7840	0.7656	0.7507	-	-
TransNetR′ 2023	27.27M	0.8706	0.8016	0.8843	0.9073	0.8744	-	-
Nanonet-Enhanced (Ours)	132,049	0.8008	0.8142	0.8588	0.8130	0.8215	16.768	0.9402
Nanonet-Enhanced with CRF (Ours)	132,049	0.8060	**0.8188**	0.8591	0.8213	0.8244	7.1884	**0.9415**
Nanonet-Enhanced with TTA (Ours)	132,049	0.7981	0.8144	0.8519	0.8219	0.8151	4.4873	0.9397
Nanonet-Enhanced with CRF and TTA (Ours)	132,049	0.8005	0.8168	0.8530	0.8263	0.8168	3.2622	0.9404

Bold indicate the best scores of one of our proposed models as compared to other state-of-the-art models: Enhanced Nanonet, Enhanced Nanonet with CRF, Enhanced Nanonet with TTA, and Enhanced Nanonet with CRF and TTA.

**TABLE 2:** Performance evaluation and comparison on Endotect 2020 Dataset.

Methods	Parameters	DSC	mIoU	Recall	Precision	F2	FPS	Accuracy
ResUNet (GRSL’18)	8,227,393	0.6640	0.5408	0.7510	0.6841	0.6943	-	0.9075
ResUNet++ (ISM’19)	4,070,385	0.6940	0.5838	0.8797	0.6951	0.7597	-	0.8841
Nanonet-A	235,425	0.7508	0.6466	0.8238	0.7744	0.7773	-	0.9255
Nanonet-B	132,049	0.7362	0.6238	0.8109	0.7532	0.7646	-	0.9252
Nanonet-C	36,561	0.7001	0.5792	0.8000	0.7159	0.7380	-	0.9091
Nanonet-Enhance d (Ours)	132,049	0.6858	0.7153	0.8866	0.6290	0.7654	17.384	0.8873
Nanonet-Enhanced with CRF (Ours)	132,049	0.6962	0.7236	0.8878	0.6437	0.7719	7.2187	0.8906
Nanonet-Enhanced with TTA (Ours)	132,049	0.7089	0.7338	0.8969	0.6595	**0.7830**	4.4763	0.8944
Nanonet-Enhanced with CRF and TTA (Ours)	132,049	0.7164	**0.7402**	**0.8978**	0.6706	0.7876	3.2771	0.8969

Bold indicate the best scores of one of our proposed models as compared to other state-of-the-art models: Enhanced Nanonet, Enhanced Nanonet with CRF, Enhanced Nanonet with TTA, and Enhanced Nanonet with CRF and TTA.

**TABLE 3:** Performance evaluation and comparison on Kvasir-Instrument.

Methods	Parameters	DSC	mIoU	Recall	Precision	F2	FPS	Accuracy
UNet (Baseline)	-	0.9158	0.8578	0.9487	0.8998	0.9320	-	0.9864
DoubleUnet (Baseline)	-	0.9038	0.8430	0.9275	0.8966	0.9147	-	0.9838
ResUNet++ (ISM’19)	4,070,385	0.9140	0.8635	0.9103	0.9348	0.9140	-	0.9866
Nanonet-A	235,425	0.9251	0.8768	0.9142	0.9540	0.9251	-	0.9887
Nanonet-B	132,049	0.9284	0.8790	0.9205	0.9482	0.9284	-	0.9875
Nanonet-C	36,561	0.9139	0.8600	0.9037	0.9452	0.9139	-	0.9863
Nanonet-Enhanced (Ours)	132,049	0.8715	0.8910	0.8867	0.8750	0.8775	17.021	0.9792
Nanonet-Enhanced with CRF (Ours)	132,049	0.8715	**0.8944**	0.8852	0.8848	0.8782	7.1580	0.9800
Nanonet-Enhanced with TTA (Ours)	132,049	0.8610	0.8847	0.8666	0.8717	0.8619	4.4631	0.9782
Nanonet-Enhanced with CRF and TTA (Ours)	132,049	0.8636	0.8874	0.8638	0.8798	0.8614	3.2752	0.9788

Bold indicate the best scores of one of our proposed models as compared to other state-of-the-art models: Enhanced Nanonet, Enhanced Nanonet with CRF, Enhanced Nanonet with TTA, and Enhanced Nanonet with CRF and TTA.

**TABLE 4:** Cross-dataset performance evaluation and comparison on Kvasir-SEG as training data.

Test set	Methods	DSC	mIoU	Recall	Precision	F2	Accuracy
CVC-ClinicDB	Enhanced Nanonet	0.6838	0.7518	0.6680	0.8580	0.6653	0.9371
	Enhanced Nanonet with CRF	0.6850	0.7535	0.6659	0.8617	0.6648	0.9372
	Enhanced Nanonet with TTA	0.7002	0.7647	**0.6695**	0.8927	**0.6742**	**0.9392**
	Enhanced Nanonet with CRF and TTA	**0.7005**	**0.7654**	0.6671	**0.8985**	0.6732	0.9391
CVC-ColonDB	Enhanced Nanonet	0.5956	0.7321	0.5792	0.7910	0.5818	0.9705
	Enhanced Nanonet with CRF	**0.5962**	**0.7328**	0.5773	0.8226	0.5808	**0.9707**
	Enhanced Nanonet with TTA	0.5683	0.7218	0.5598	0.8063	0.5589	0.9697
	Enhanced Nanonet with CRF and TTA	0.5675	0.7221	0.5585	**0.8684**	0.5578	0.9698
CVC-300	Enhanced Nanonet	0.5514	0.7153	0.5370	0.8395	0.5352	0.9782
	Enhanced Nanonet with CRF	0.5422	0.7149	0.5311	0.8839	0.5302	0.9785
	Enhanced Nanonet with TTA	0.5514	0.7201	0.5308	0.8654	**0.5344**	0.9791
	Enhanced Nanonet with CRF and TTA	0.5421	**0.7178**	0.5243	**0.8893**	0.5273	**0.9791**

Bold indicate the best scores of one of our proposed models as compared to other state-of-the-art models: Enhanced Nanonet, Enhanced Nanonet with CRF, Enhanced Nanonet with TTA, and Enhanced Nanonet with CRF and TTA.

**FIGURE 5:**
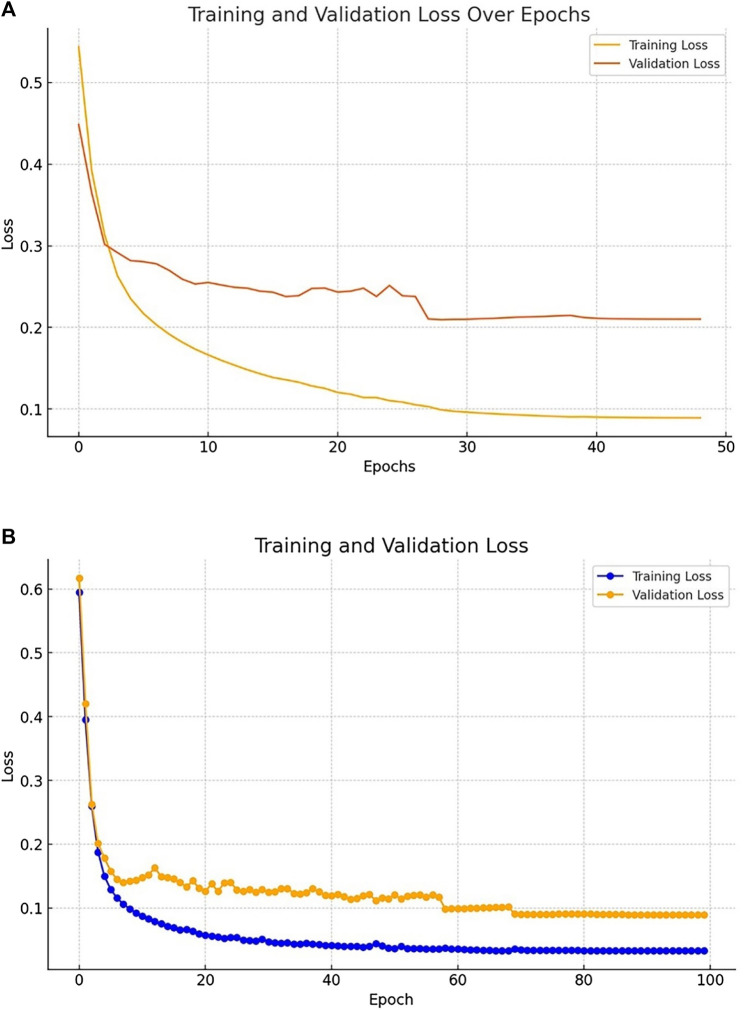
**(A)** Losses evaluated on the Kvasir-SEG dataset **(B)** Losses evaluated on the Kvasir-Instrument dataset.

Figures 5A, B shows the training and validation curves for Kvasir-SEG and Kvasir-instrument dataset.

### 4.1 Results on Kvasir-SEG dataset

**FIGURE 6:**
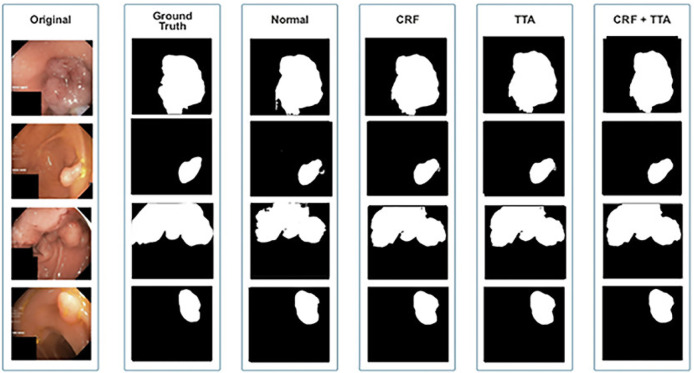
Qualitative results of Enhanced Nanonet on Kvasir-SEG dataset.

**FIGURE 7:**
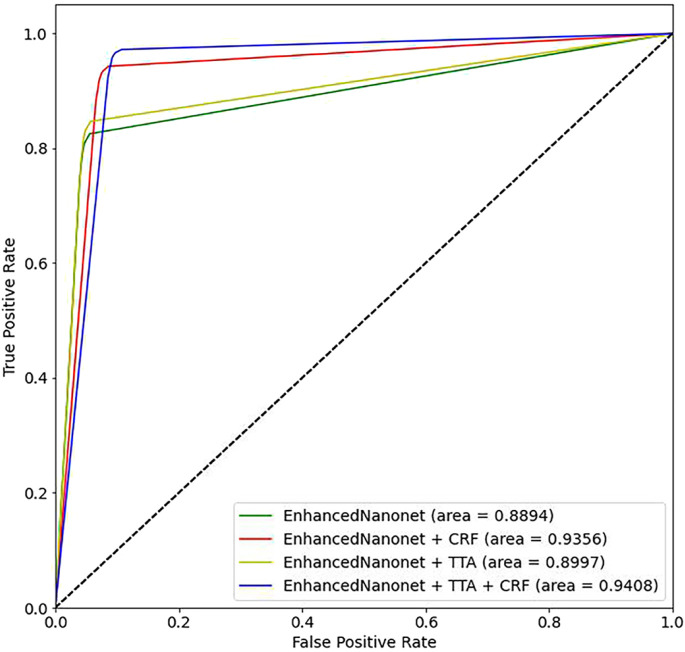
Proposed model ROC curves on the Kvasir-SEG dataset.

Figure 6 and Table 1 depict Qualitative and quantitative comparisons of the Kvasir-SEG dataset results. As demonstrated by the qualitative results (Figure 6) and quantitative results (Table 1), the proposed Enhanced-Nanonet model outperforms almost all recent SOTA methods and achieves the highest mIoU, Recall, Precision, F2, and Accuracy for the Kvasir-SEG dataset. The proposed model’s ROC curve for the Kvasir-SEG dataset is shown in Figure 7. Table 1 shows that the combination of the proposed Enhanced Nanonet with CRF achieves mIoU 0.8188, which is 18.25% higher than SOTA (Jha et al., 2019), 9.06% better than SOTA (Jha et al., 2021) and 20.82% better than SOTA (Hou et al., 2016). Similarly, other evaluation metrics (Recall, Precision, F2, and Accuracy) surpass other advanced methods regarding results mentioned in Table 1. The DSC scores of all three proposed plans on the Kvasir-SEG dataset are good. The Enhanced-Nanonet model has demonstrated a significant improvement over all baseline architectures on the Kvasir-SEG dataset, as measured by all performance evaluation metrics. The enhanced results show the significance of using the right data augmentation strategies, TTF, CRF, and their combination.

### 4.2 Results on endotect 2020 dataset

**FIGURE 8:**
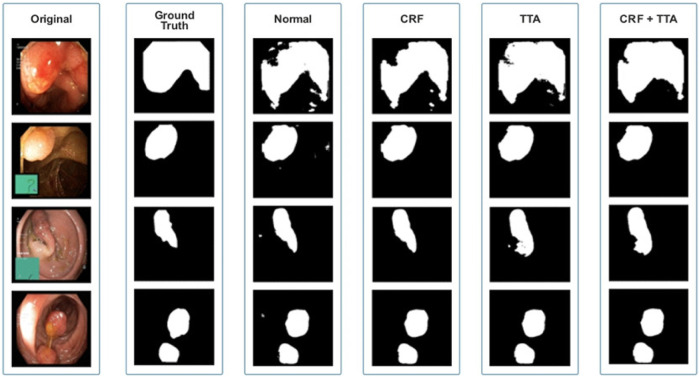
Qualitative results of Enhanced Nanonet on Endotect dataset.

**FIGURE 9:**
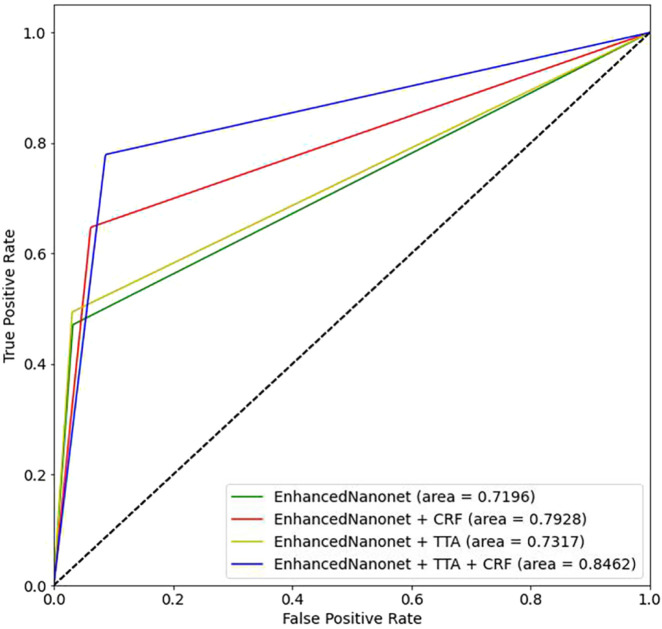
Proposed model ROC curves on the Endotect 2020 Challenge dataset.

Figure 8 and Table 2 depict qualitative and quantitative comparisons of results on the Endotect 2020 dataset. Additional experiments were performed for in-depth analysis of automatic colorectal cancer segmentation. Figure 9 shows the ROC curve for the proposed model on the Endotect Challenge 2020 dataset. Table 2 shows that the combination of the proposed Enhanced Nanonet with CRF and TTA achieves mIoU 0.7402, which is 19.94% higher than SOTA (Hou et al., 2016), 15.64% better than SOTA (Jha et al., 2019) and 9.36% better than SOTA (Jha et al., 2021). Similarly, other evaluation metrics (Recall and F2) produce superior outcomes than other cutting-edge techniques mentioned in Table 2. The DSC score, precision and accuracy of all three proposed methods on the Endotect 2020 dataset are pretty good. As shown in quantitative (Table 2) and qualitative results (Figure 8), the proposed Enhanced-Nanonet model achieves remarkable results regarding mIoU, Recall, and F2 scores compared with recent deep learning models for the Endotect dataset. Therefore, Table 2 and Figure 8 show the advantage of using TTA and its combination with CRF on the Endotect 2020 dataset.

### 4.3 Results on Kvasir-instrument dataset

**FIGURE 10:**
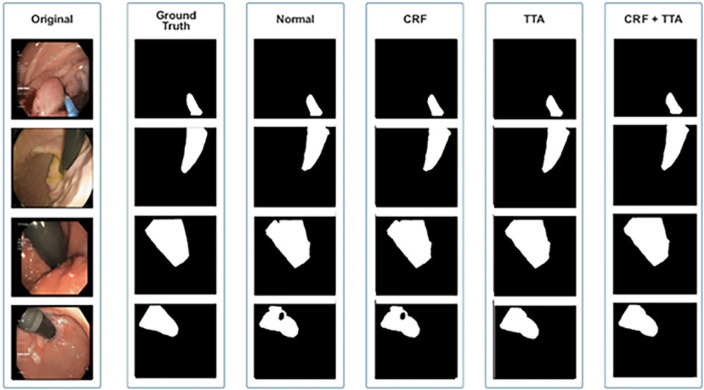
Qualitative results of Enhanced Nanonet on Kvasir-Instrument dataset.

**FIGURE 11:**
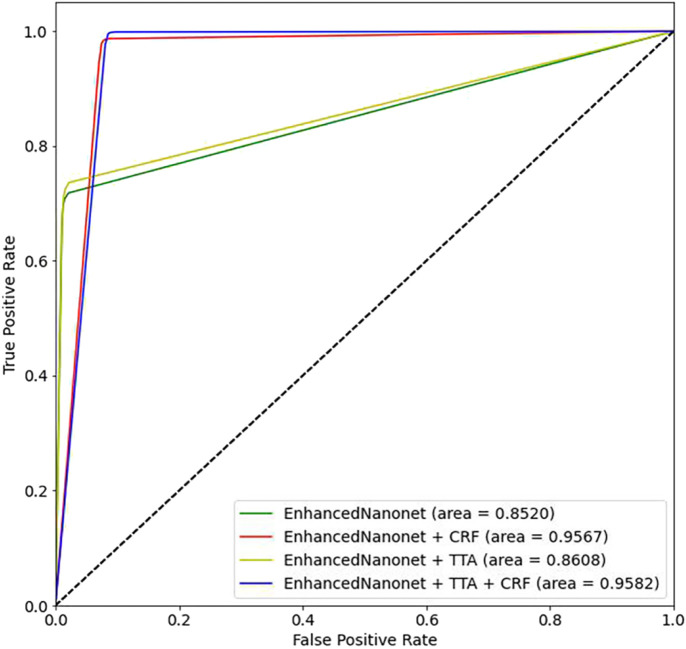
Proposed model ROC curves on the Kvasir-Instrument.

Figure 10 and Table 3 depict qualitative and quantitative comparisons of results on the Kvasir-Instrument dataset. The enhanced Nanonet model is trained with the Kvasir-Instrument dataset for just nine epochs to reduce training time and achieve better results. Figure 11 shows the ROC curve for the proposed model on the Kvasir-Instrument dataset. Table 3 demonstrates that the proposed model combination with CRF achieves mIoU 0.8944, which is 3.66% higher than SOTA (Ronneberger et al., 2015), 5.14% better than SOTA (Jha et al., 2020), 3.09% better than SOTA (Jha et al., 2019) and 1.76% better than SOTA (Jha et al., 2021). Similarly, other evaluation metrics achieve competitive results mentioned in Table 3. It can be observed that in just nine epochs, the model achieves promising results in comparison with recent SOTA computer vision methods. The enhanced-Nanonet model trained on the Kvasir-Instrument dataset has outperformed all baseline architecture in terms of mIoU, as shown in Table 3, which plays a crucial role in colorectal cancer detection. With hyperparameter tuning, data augmentation, and applying CRF and TTA, results have significantly improved.

### 4.4 Evaluation of cross-dataset results on Kvasir-SEG dataset

The model was trained using the Kvasir-SEG dataset, and its cross-dataset performance was assessed using three additional distinctive, independent datasets. The proposed model’s cross-dataset generalizability outcomes are shown in Table 4, both when used independently and in conjunction with CRF and TTA approaches. On various polyp image datasets, the proposed models yield an average best Dice Similarity Coefficient (DSC) and mean Intersection over Union (mIoU) of 0.6070 and 0.7343 trained on Kvasir-SEG. Results indicate that the suggested combination approaches exhibit competitive performance. The combined use of Enhanced Nanonet with TTA demonstrates better performance among the various datasets. The model consistently performs well on datasets from clinical centers that have never been seen before, based on data from three datasets. This shows a better generalization ability than the latest methods. Our model was trained using 1,000 polyp images and a limited number of parameters and still achieved better generalization results on unseen datasets.

## 5 Discussion

Enhanced Nanonet model is developed based on NanonetB, which significantly improves upon the original Nanonet architecture. The proposed model uses MobileNetV2, pre-trained on ImageNet, as the encoder, followed by three custom decoder blocks. MobileNetV2 includes inverted residuals and linear bottlenecks, which reduce the number of parameters while maintaining high performance. This architecture choice ensures that the model is both lightweight and capable of extracting rich feature representations from the input images. A modified residual block between the encoder and decoder enhances feature extraction and overall performance. The encoder leverages MobileNetV2’s efficiency to capture rich features, while the modified residual block effectively integrates these features, preserving essential information.

The decoder, composed of three blocks built with modified residual blocks, reconstructs the image with high accuracy. The custom decoder in Enhanced Nanonet is specifically designed to work with the MobileNetV2 encoder. The decoder includes optimized layers that focus on preserving spatial resolution and enhancing feature refinement without adding unnecessary complexity or parameters. This careful design ensures that the segmentation accuracy is high while keeping the model lightweight. This flow ensures that the model captures, processes, and utilizes contextual information effectively, resulting in precise segmentation outputs. It incorporates extensive data augmentation, CRF for refined boundary prediction, and TTA for robust inference. This combination has not been previously explored (as per the literature review) in the context of lightweight, real-time segmentation models, making the proposed approach unique. One of the key contributions of this study is the focus on creating a more generalized and lightweight model that can be easily integrated into clinical practice. Unlike many existing deep learning models that are computationally intensive and require substantial resources, the proposed Enhanced Nanonet model achieves high accuracy with minimal computational overhead. This is particularly important for real-time applications in resource-limited settings. To ensure the robustness and generalizability of the proposed model, we conducted thorough evaluations on six publicly available datasets. Our extensive cross-dataset evaluation demonstrates that the proposed model performs consistently well across diverse datasets, which is a critical aspect of developing models suitable for real-world clinical applications.

The qualitative and quantitative results suggest that the proposed Enhanced Nanonet model with CRF and TTA, along with their combination, outperforms and, in some cases performs very near to other sophisticated deep learning networks in terms of mIoU, DSC, and additional evaluation metrics on the same and cross-dataset. The qualitative results are shown in Figures 6, 8, 10. Starting from the left in figures, the first, second, third, fourth, fifth, and sixth columns show the image, ground truth, Enhanced Nanonet model, Enhanced Nanonet with CRF, with TTA, and lastly, CRF and TTA combined. Four sample images from each dataset are presented. One of the significant strengths of our work is the effective utilization of parameters. Table 1 clearly shows that the proposed Enhanced Nanonet models with CRF and TTA, along with their combination, use 62 times fewer parameters than ResUNet (GRSL’18) and 207 times lesser than TransNetR′ 2023, also achieve better and competitive results in terms of mIoU, DSC and other evaluation metrics on Kvasir-SEG dataset. While acknowledging existing studies and module designs, the study includes a thorough comparative analysis, showcasing the superior performance of the Enhanced Nanonet model against existing state-of-the-art methods. The reported Dice score of 0.8060 and mean Intersection over Union (IoU) of 0.8188 Kvasir-SEG dataset with just 132,049 parameters underscore the effectiveness of the proposed method.

The qualitative results across various medical datasets suggest the proposed model can generate accurate segmentation maps for diverse lesion (polyps) classes with minimal parameters. Also, it demonstrates a notable proficiency in effectively segmenting smaller, flat, or sessile polyps. Results also depict that our model produces good segmentation results on small, medium, and large-size polyps (see Figures 6, 8, 10), often overlooked during the endoscopic examination, thus making it well-suited for developing an optimal CADx polyp detection system. Specifically, the proposed NanonetB deep learning architecture is extended by applying data augmentation and integrating CRF, TTA, and their combination. This enhancement has led to marked improvements in segmentation performance across multiple datasets, including those specifically containing sessile and flat polyps. Enhanced Nanonet with CRF and TTA produces excellent segmentation maps for all types of polyps in comparison with other techniques mentioned in the results section. This is a prominent strength in our work, making it suitable for clinical testing. The cross-data test is valuable for assessing a model’s generalization capabilities. This study represents an effort to improve segmentation techniques generalizability. Achieving generalizability entails training the model on one dataset, testing it on several additional public datasets from various centers, and using different scope manufacturers. Tackling this issue requires using multicenter data that is not part of the sample to evaluate the effectiveness of the techniques that have been created. This study is a step in highlighting concerns related to method interpretability and prompts inquiries about the domain adaptability and generalizability of supervised methods in the broader setting. Additionally, an in-depth examination of the cross-dataset generalizability involved training on Kvasir-SEG, followed by testing on three distinct datasets, affirming the adaptability of the proposed model with CRF, TTA, and their combination method in cross-dataset evaluations. Thus, employing post-processing techniques like CRF and TTA enhances the colonoscopy image segmentation results, by utilizing lightweight models with a pre-trained encoder.

Several challenges associated with our work are the quality of bowel preparation during colonoscopy, varying morphology, and the angle of cameras impacting the deep learning model’s overall performance. There is also some variation of decisions between endoscopists for some images. Despite facing challenges in generating satisfactory segmentation maps for these images, the proposed Enhanced Nanonet model with CRF and TTA performs significantly better than the original Nanonet model with fewer parameters and surpasses other state-of-the-art algorithms. It has been noticed that batch size, the number of filters, optimizers, and loss functions significantly impact results. One of the limitations of our work is that to reduce complexity, a 256 × 256 resizing is applied to the training images, which leads to information loss and impacts the overall effectiveness of the model. We have extensively optimized the code to the best of our knowledge and experience. Moreover, further optimizations may exist, which could also impact the results of the architectures. However, the Enhanced Nanonet model with CRF and TTA provides robust solutions for real-time applications. Compared to other SOTA approaches, it yields outstanding results with fewer parameters.

## 6 Conclusion

This work proposes novel lightweight Enhanced Nanonet models (with CRF, TTA, and their combination) for efficient and precise segmentation of polyps found in colonoscopy examination. Data augmentation and post-processing techniques (CRF and TTA) have been applied on NanonetB to improve results. The proposed Enhanced Nanonet models are trained and validated with and without CRF and TTA techniques on six different datasets, achieving higher performance and generalizability. The results show improved results as compared to other state-of-the-art (SOTA) algorithms, producing accurate semantic predictions. The proposed model’s cross-data generalizability aims to address and advance the development of semantic segmentation models with broad applicability in automatic polyp segmentation. It involves training on the Kvasir-SEG dataset, followed by testing on three independent datasets, affirming the robustness of the proposed model with CRF and their combination in cross-dataset evaluations. The main architecture of Nanonet in the proposed model uses pre-trained MobileNetV2 and modified residual blocks. The depth-wise separable convolution allows the model to achieve higher performance with less trainable parameters. The proposed method’s strength lies in effectively detecting smaller and flat polyps, that are normally overlooked during colonoscopy examinations. Additionally, the proposed model can identify polyps that might pose challenges for endoscopists to detect without thorough investigations. Hence, the NanonetB architecture, coupled with the CRF and TTA and their combination, effectively addresses overlooked polyps. The proposed deep learning enhanced Nanonet model in clinical systems can integrate with real-time endoscopic hardware devices because of fewer parameters, more generalizability, competitive accuracy, and low latency. The proposed Enhanced Nanonet technique may also offer a firm baseline in developing clinically applicable methods for further investigations. In the future, we aim to improve the speed in terms of frames-per-second (FPS) and the model trails, utilization in actual clinical settings.

## Data Availability

The original contributions presented in the study are included in the article/Supplementary Material, further inquiries can be directed to the corresponding author.
